# Abdominal Pseudohernia Caused by Zoster Sine Herpete

**DOI:** 10.7759/cureus.73728

**Published:** 2024-11-15

**Authors:** Koji Tajima, Toshiki Mushino

**Affiliations:** 1 Internal Medicine, Kokuho Nokami Kosei Sogo Hospital, Kimino, JPN; 2 Community Medical Support Center, Wakayama Medical University, Wakayama, JPN

**Keywords:** constipation, muscle thinning, pseudohernia, ultra sound, zoster sine herpete

## Abstract

Abdominal pseudohernia is a rare clinical condition characterized by an abnormal bulging of the abdominal wall. We present a 56-year-old male patient diagnosed with abdominal pseudohernia caused by zoster sine herpete (ZSH). The patient initially presented with a right abdominal wall bulging at the Th10 dermatome area. The patient did not experience any pain or rash at the site, and the ultrasonography (US) did not reveal any tumor or hernia. Computed tomography (CT) was taken in another hospital, but it failed to provide a diagnosis. During the second visit, we performed standing ultrasonography and detected the thinning of the abdominal muscle. To diagnose abdominal pseudohernia, the key point is to detect the muscle thinning between the affected site and the healthy side.

## Introduction

Reactivation of herpes zoster (HZ) is a common condition experienced in the elderly and immunocompromised patients. HZ remains latent in the dorsal root ganglion after initial infection during childhood. After initial infection, approximately 30% of people are affected by HZ reactivation in their lifetime [1]. Clinical symptoms of HZ reactivation appear in three stages: pre-eruptive, acute exudative, and chronic [2]. The patient in the pre-eruptive stage has burning pain within the affected dermatome. In the acute phase, multiple painful rashes, such as vesicles, ulcers, and eschars, are mixed. The acute eruptive phase can last two to four weeks. The chronic phase is characterized by severe pain, dysesthesias, or paresthesia that lasts more than four weeks. This is the most common complication of HZ reactivation, called postherpetic neuralgia. This reduces the quality of life due to severe pain that can persist even after the rash diminishes. Other complications include ophthalmic involvement, neurological complications, and stroke [1].

Abdominal pseudohernia is an abdominal wall bulge caused by various conditions, such as diabetes mellitus [3], spinal disk hernia [4-5], surgical injury [6], and rib fracture [7]. These conditions lead to an atrophic change in the abdominal wall muscle. Abdominal pseudohernia resulting from HZ reactivation involves motor neurons of the abdominal muscles; that is, HZ reactivation leads to muscle atrophy and results in abdominal pseudohernia. This condition, a rare manifestation of HZ reactivation, often goes unnoticed or misdiagnosed. It is not difficult to diagnose when the patient has a rash, but it can be challenging to diagnose when the patient does not. "Zoster sine herpete (ZSH)" refers to the condition where patients present highly suggestive symptoms and signs of HZ reactivation without a rash [8].

ZSH was termed by Lewis for the cases in which clinicians highly suspect the HZ reactivation from clinical symptoms and courses but without a rash [8]. Lewis proposed important points to suspect ZSH: (i) unilateral segmental pain of a sclerotomal or dermatomal type, or both, with complete recovery in a few weeks; (ii) certain painful unilateral muscular paresis of obscure origin; (iii) unilateral segmental pain associated with certain visceral disturbances of short duration and complete resolution; (iv) unilateral ophthalmic neuralgia with involvement of the eyeball, or with paresis of the ocular muscles, or both; (v) unilateral otalgia without evidence of middle-ear disease and associated with facial palsy, hyperacusis, or loss of taste sensation on the anterior two-thirds of the tongue; (vi) cases presenting as acute labyrinthitis, or Menier’s syndrome, with evidence of the involvement of adjacent nerves, particularly the seventh cranial nerve; (vii) unilateral paralysis of the origin, especially when associated with otalgia or with an inflammatory reaction in, or around, the entrance to the larynx [8]. However, even if we follow these points, it is necessary to monitor the clinical course of the disease, and it is suggested that ZSH may not be diagnosed during the acute phase.

To diagnose abdominal pseudohernia, two points need to pass: (i) to prove HZ reactivation and (ii) to rule out other diseases that cause abdominal bulges. To rule out other diseases like abdominal hernia or tumors, any imaging modality must be used. A computed tomography (CT) scan can examine the whole abdomen. Ultrasonography (US) can examine the partial area in the abdomen. In terms of spatial resolution, the US is generally better than CT.

We report a rare case of a 56-year-old man who presented to our clinic with a bulging abdominal wall without any skin rash. We clinically diagnosed the patient with abdominal pseudohernia due to ZSH, which is the reactivation of herpes zoster without a skin rash. The key points of this case are the time course of his symptoms and the findings of the US.

## Case presentation

A 56-year-old male patient presented to our clinic with a three-day history of lower back pain. He had gone surfing five days earlier. He had experienced similar pain after previous surfing sessions. He initially took over-the-counter pain medications. However, the pain persisted. The patient had a history of type 2 diabetes mellitus, hypertension, hyperuricemia, dyslipidemia, and fatty liver, all of which were well controlled with metformin, vildagliptin, amlodipine besylate, enalapril maleate, atorvastatin, and febuxostat. He had never had HZ reactivation. He did not remember whether he had a varicella zoster infection in childhood. He was neither a smoker nor a social drinker.

The pain did not worsen with extension, flexion, or rotation of the lower back. There was no associated radiating pain to the lower limbs, spasticity, or bladder or rectal dysfunction. The patient presented mild tenderness on the right side of the spinous process of the L4 lumbar vertebra. We performed a US scan and administered US-guided fascia hydrorelease with dibucaine hydrochloride.

**Figure 1:**
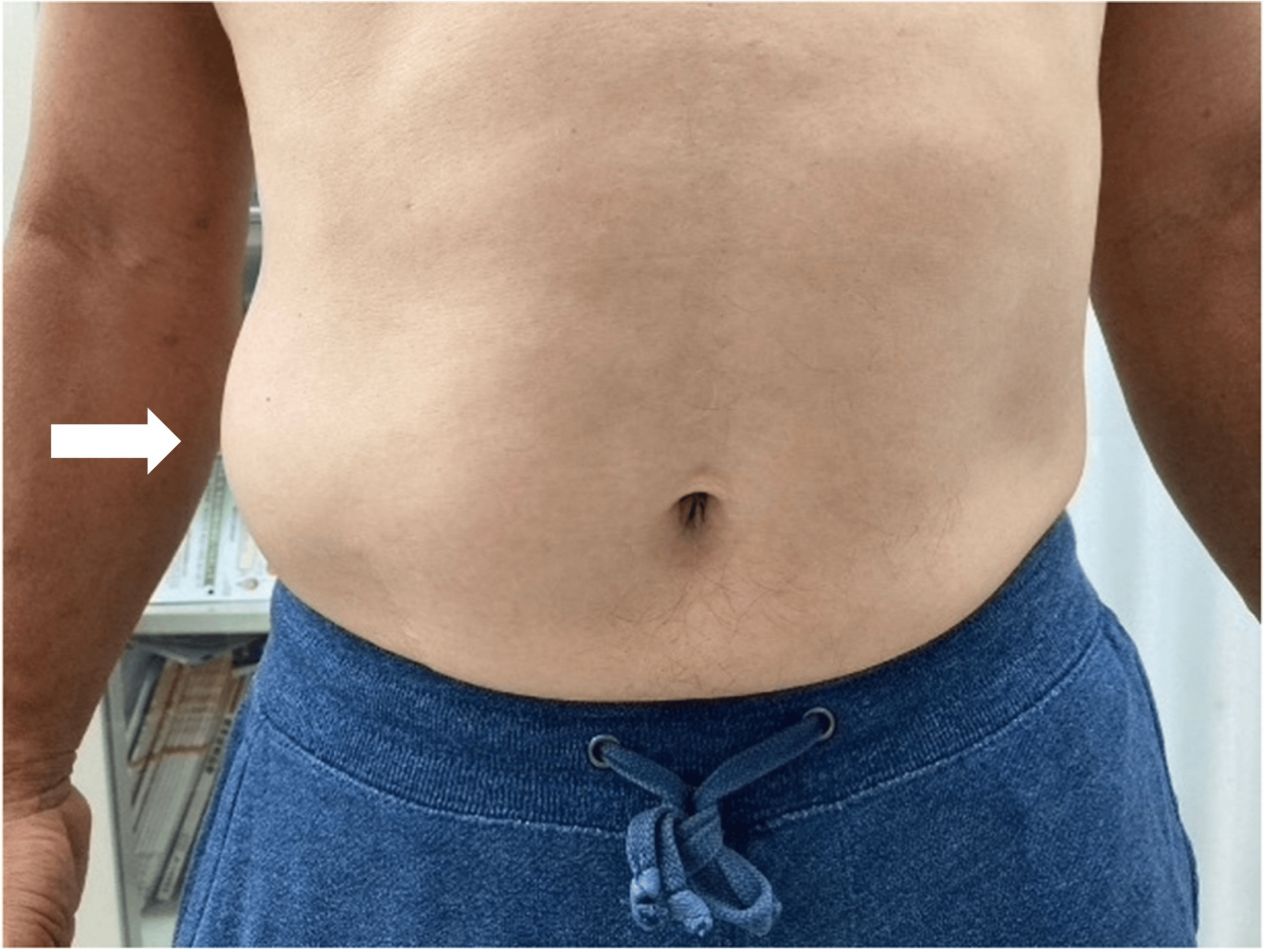
Image of the abdominal wall bulge. The patient noticed a painless abdominal bulge in the right flank without a rash (arrow).

**Figure 2:**
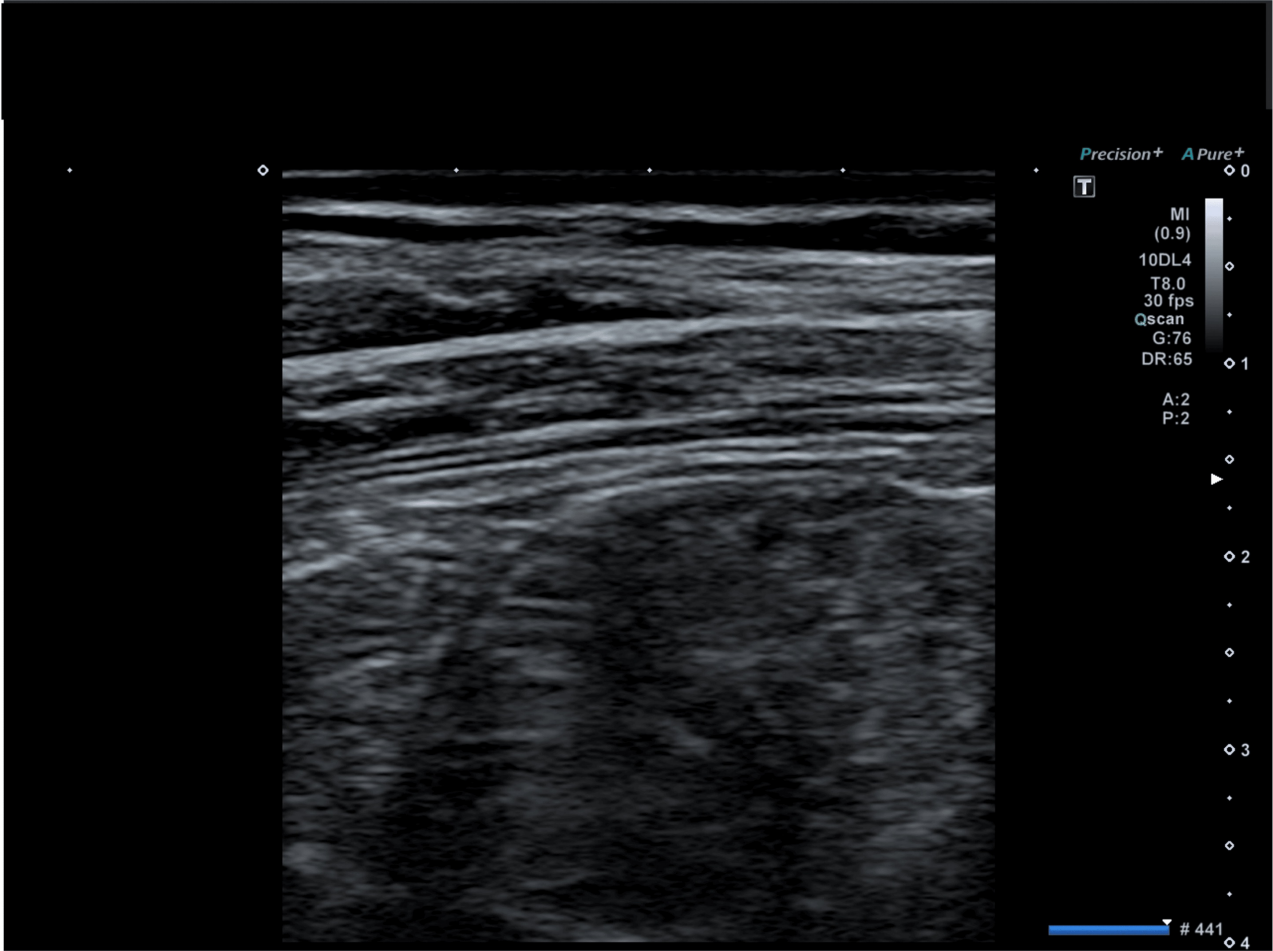
Ultrasonography image. A mass or tumor was not detected at the affected site.

The day after the initial visit, the patient returned to the clinic due to the right abdominal wall bulging in the Th10 dermatome area (right flank), which was a new symptom (Figure 1). The bulge was about the size of an adult's palm. The US in the supine position detected no tumor or hernia, nor was there any pain or rash at the site (Figure 2). The sensation between the affected and healthy sides was similar.

**Figure 3:**
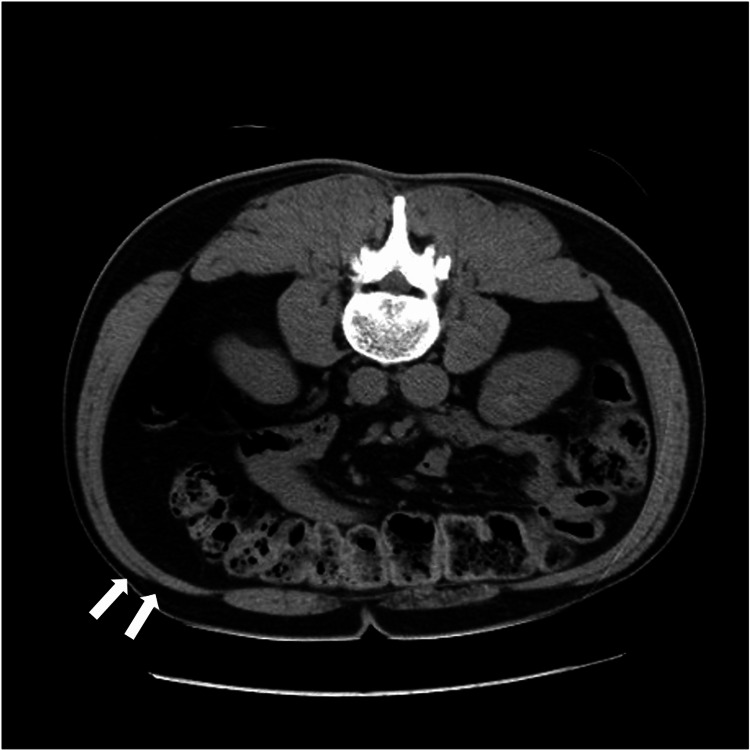
Computed tomography image. A mass or hernia was not detected, and the patient’s diagnosis was unclear. However, a CT scan reveals retrospectively slight muscle atrophy at the right flank compared with the left flank (arrow).

**Figure 4:**
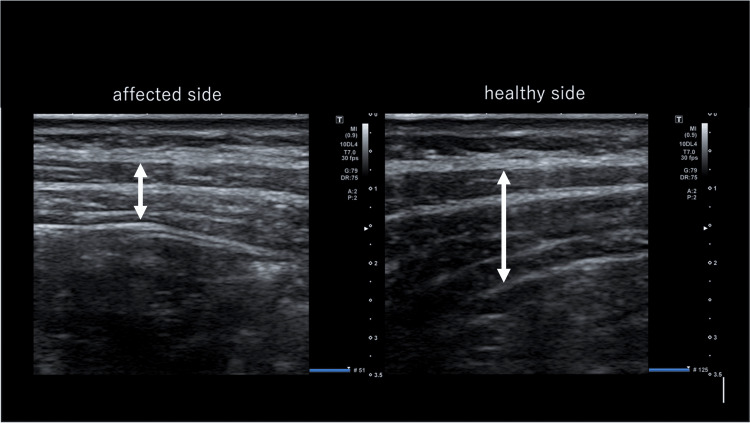
Ultrasonography image. Thinning of the abdominal muscles was observed on the affected side compared with the healthy side. The arrow indicates the muscle layer.

**Figure 5:**
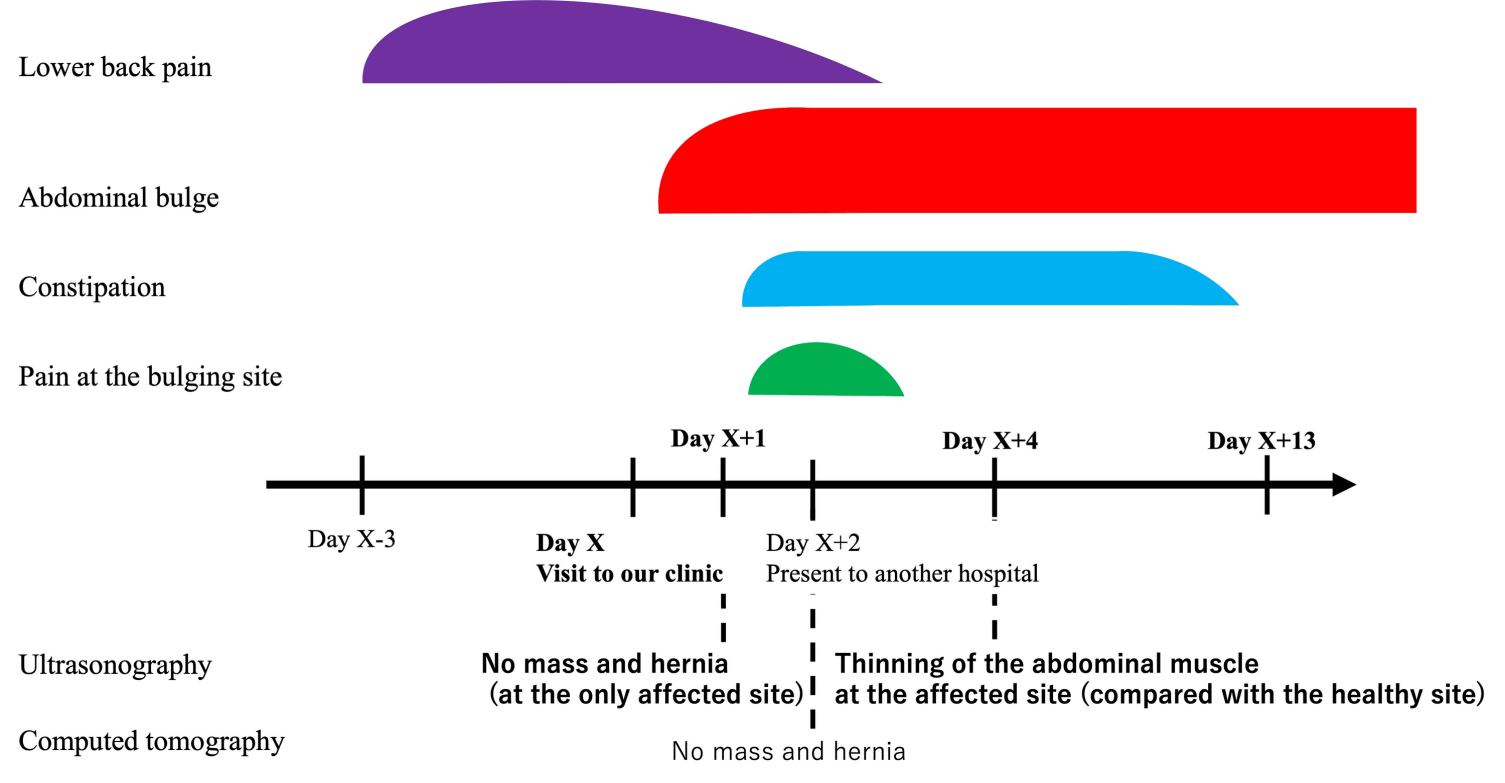
Summary of the patient’s clinical course and image findings. He first presented to our clinic with lower back pain on day X. On day X+1, the patient revisited our clinic due to an abdominal bulge at the right flank. Thereafter, because the patient noticed the pain at the bulging site and constipation, he presented to another hospital on day X+2. However, the diagnosis was unclear. On day X+4, the patient came to our clinic due to the remaining abdominal bulge. An ultrasonography revealed the thinning of the abdominal muscle at the affected site compared with the healthy site. Based on these findings and clinical course, the patient was clinically diagnosed with abdominal pseudohernia caused by zoster sine herpete. The bold words represent the days and image findings of the patients who came to our clinic.

On the third day after the second visit, the patient complained of constipation. He also experienced stabbing pain in the bulged area of the abdominal wall. However, the pain disappeared the next day. He visited the emergency department of another hospital. A CT scan was performed (Figure 3). Nevertheless, the cause of these symptoms remained unknown. His lower back pain had resolved by the seventh day, but the abdominal wall bulge and constipation remained. Subsequently, he revisited our clinic. A US examination of the abdominal wall revealed thinning of the external and internal oblique muscles and transversus abdominis muscle, particularly the internal oblique muscle at the affected site (Figure 4). A summary of the patient’s clinical course and image findings is shown in Figure 5. The patient's reported lower back pain was near the L4 area, which did not match the Th10 dermatome area presenting with abdominal bulge and, therefore, was determined not to be a symptom of reactivation of HZ. In addition, a spinal disk hernia was one of the differential diagnoses of abdominal pseudohernia; however, the dermatome with the lower back pain was inconsistent with the abdominal bulge. Moreover, type 2 diabetes mellitus, which the patient had, was well controlled with medications. The patient had never had any injury or operations that might cause the abdominal bulge. We ruled out spinal disk hernia, diabetes mellitus, injury, and operation as the cause of the abdominal bulge. Based on the clinical course, which suspected the HZ reactivation, such as the pain and constipation, and image findings that could rule out any mass lesions or hernia, the patient was clinically diagnosed with abdominal pseudohernia caused by ZSH. The constipation seemed to be a neurological complication of the gastrointestinal system due to HZ reactivation. The patient preferred monitoring without antiviral agents because he had no pain.

Regular follow-up of the pseudohernia was performed. The patient no longer complained of constipation after one week. Seven months later, the abdominal wall distension persisted.

## Discussion

The common manifestations of HZ reactivation include rash and neuralgia along the dermatome. In addition, gastrointestinal (GI) complications, including constipation and muscle atrophy caused by neurological dysfunction of the visceral or motor nerves, are other manifestations of HZ reactivation [9]. However, some patients do not exhibit the typical skin rash, a condition known as ZSH. ZSH is challenging to diagnose because of the lack of skin lesions. However, clinicians should be aware of the diagnosis and management of ZSH because delayed treatment can lead to persistent postherpetic neuralgia, which significantly affects quality of life.

Abdominal pseudohernia is an abdominal wall bulge caused by an atrophic change in the abdominal wall muscle. The diagnosis of abdominal pseudohernia is based on diagnostic imaging to rule out true hernias and tumors.

Furthermore, it is important to obtain an accurate diagnosis to detect thinning of the abdominal wall muscles, such as the external and internal oblique muscles and the transverse abdominis muscle, with imaging, which reflects denervation and nerve palsy caused by HZ reactivation. However, only 20.6% of patients present with these imaging features [10]. In this case, the physician could not point out the muscle atrophy with CT. However, a CT scan could retrospectively detect slight atrophy of the right abdominal wall. On the other hand, due to its excellent spatial resolution and the comparison with the healthy side, the US was useful for confirming the diagnosis of this patient. While CT ruled out a true hernia or abdominal mass, US was more sensitive in detecting muscle thinning, crucial for diagnosing pseudohernia. During the first visit, US was performed on the affected side only to examine for a true hernia or mass. Therefore, thinning of the abdominal wall muscle was not detected. On the second visit, the diagnosis was made by comparing the thickness of the muscles on the affected and healthy sides. Therefore, it is important to assess the difference in the muscles between the left and right sides using the US, and the slight difference may be missed by CT, like in this case. Moreover, the US may be more effective in detecting slight differences in muscle thickness due to the high resolution of its imaging, and its repeatability is also a significant advantage of the US.

In this case, a diagnosis of pseudohernia was made based on the presence of constipation and US findings. GI complications, including constipation, are associated with the involvement of extrinsic autonomic or motor neurons, including the intestinal muscularis propria and myenteric plexi [11]. Approximately 19.4% of patients with pseudohernia present with GI complications induced by HZ reactivation [12]. Clinicians should be cautious when taking a medical history related to GI complications.

Abdominal pseudohernia caused by HZ reactivation is an extremely rare condition [13]. The initial characteristics of abdominal pseudohernia attributed to HZ reactivation are pain (typically severe burn pain) and skin rash, and the rash generally precedes abdominal bulging [12]. In some cases, pain persists after the rash has diminished. However, in a few cases, the abdominal wall bulge precedes the rash or occurs in the absence of a rash, as in this case [12]. Moreover, in some cases, the patient only presents with mild pain or an itching sensation; thus, it is challenging to consider HZ reactivation as a differential diagnosis. In this patient, pseudohernia was present without rash, and the patient experienced right flank stabbing pain for only one day. Although this is an atypical clinical course of HZ reactivation, clinicians should be cautious regarding the various manifestations of HZ reactivation. Conservative management is the treatment of choice for patients with pseudohernia caused by HZ reactivation, and 70% of patients receiving this type of therapy have completely recovered [10]. In this case, the patient had no pain when he was diagnosed with pseudohernia due to ZSH. Therefore, the patient decided not to take any antiviral agents. 

The current report has several limitations. Direct verification of HZ or serum antibody titer was not performed. However, the other causes of abdominal pseudohernia, including diabetes mellitus, spinal disk hernia, surgical injury, and rib fracture, were ruled out with physical examinations and other appropriate tests. If the serum antibody titer was performed, the definitive diagnosis of pseudohernia due to ZSH was strengthened. As this is a case report, further studies with large sample sizes are warranted to validate our findings. In particular, future studies with more cases of pseudohernia due to HZ reactivation should be conducted to establish standard examinations and treatments.

## Conclusions

Abdominal pseudohernia is one of the rare conditions caused by HZ reactivation. This case highlights the difficulty of diagnosis for abdominal pseudohernia due to ZSH. This case report has the limitation that direct confirmation of ZSH through antibody testing was not performed. Future cases could benefit from this testing to improve diagnostic accuracy. When encountering an abdominal bulge without a rash, physicians need to consider abdominal pseudohernia due to ZSH. GI complications such as constipation may serve as a clue to this condition, as they may arise from the autonomic nerve's involvement in the reactivation of HZ. US may be more effective than CT for detecting subtle muscle changes between the affected and unaffected sides. In many cases, this condition is self-limiting; it is a treatment option not to take the antiviral agent if the patient has no pain. In addition, it is also necessary to reassure the patient.
